# Anatomical Analysis of the Gonadal Veins and Spine in Lateral Lumbar Interbody Fusion

**DOI:** 10.3390/jcm12083041

**Published:** 2023-04-21

**Authors:** Yujiro Kagami, Hiroaki Nakashima, Kotaro Satake, Kenyu Ito, Mikito Tsushima, Naoki Segi, Hiroyuki Tomita, Jun Ouchida, Yoshinori Morita, Yukihito Ode, Shiro Imagama, Tokumi Kanemura

**Affiliations:** 1 Department of Orthopedic Surgery, Konan Kosei Hospital, Konan 483-8704, Japan; 2 Department of Orthopedic Surgery, Anjo Kosei Hospital, Anjo 446-8602, Japan; 3 Department of Orthopedic Surgery, Nagoya University Graduate School of Medicine, 65 Tsurumai, Shouwa-ku, Nagoya 466-8560, Japan

**Keywords:** gonadal veins, lateral lumbar interbody fusion, degenerative scoliosis

## Abstract

Background: The current study aimed to investigate the anatomical position of the gonadal veins (GVs) from the viewpoint of spine surgery and the risk factors associated with lateral lumbar interbody fusion (LLIF). Methods: This retrospective study included 99 consecutive patients. The GV locations were divided into the ventral (V), dorsal medial (DM), and dorsal lateral (DL) sides based on lumbar disk levels on axial contrast-enhanced computed tomography images. The DM region surrounded by the vertebral body and psoas muscle had the highest risk of GV injury. The GV at each intervertebral disk level was examined in terms of laterality and sex. The patients were divided into group M (which included those with GV in the DM region at any vertebral level) and group O (which included those without GV in the DM region at any vertebral level). Then, the two groups were compared. Results: In the case of lower lumbar levels and in women, the GVs were commonly observed in the DM region. Group M had a higher incidence of degenerative scoliosis than group O and a significantly larger Cobb angle. Conclusions: We should pay close attention to the GV location on the preoperative image when using LLIF, particularly in female patients with degenerative scoliosis.

## 1. Introduction

Lateral lumbar interbody fusion (LLIF) is a surgical technique for managing lumbar spondylolisthesis and adult spinal deformity [1,2,3,4]. Using LLIF, the disc space can be reached with direct lateral interbody fusion (DLIF) and extreme lateral interbody fusion (XLIF) via the transpsoas corridor. Meanwhile, oblique lateral interbody fusion (OLIF) involves access to the disc space via the corridor between the peritoneum and psoas muscle [5]. Compared with the more traditional posterior approach, LLIF has several benefits. That is, it is associated with a shorter operative time, lower volume of intraoperative blood loss, greater indirect decompression, and higher fusion rates [6,7]. Nevertheless, there are reports of damage to retroperitoneal organs, including the nerve roots, blood vessels, intestine, and ureters [8]. LLIF requires attention to vascular injuries due to its proximity to the corridor.

**Figure 1:**
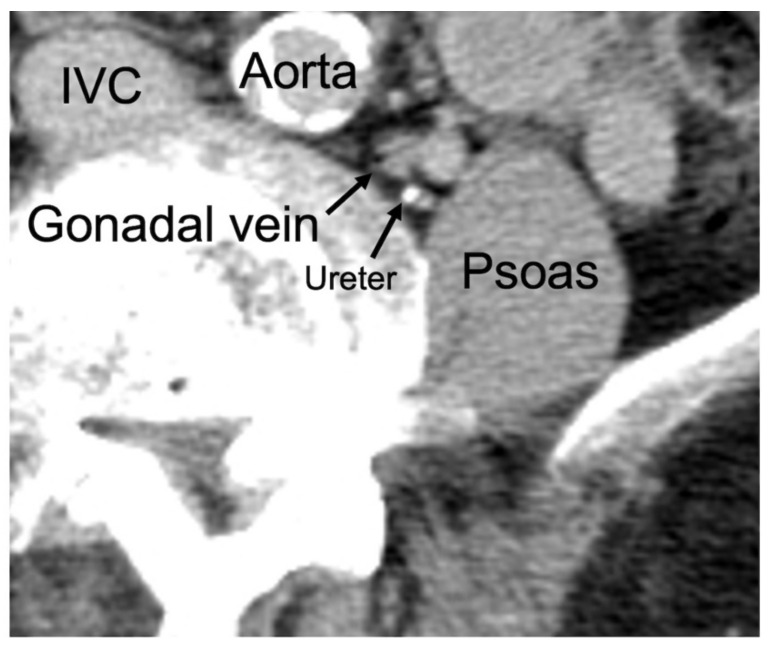
Gonadal vein between the vertebral body and iliopsoas muscle. IVC: inferior vena cava, Psoas: iliopsoas.

Among intraoperative vascular injuries, major vascular injuries could be fatal, and minor vascular injuries are associated with a longer operative time [9]. Gonadal vein (GV; testicular and ovarian vein) injury is a major vascular injury, and the incidence of ovarian vein injury was approximately 0.9% [9,10]. The incidence of ovarian vein injury is low. However, it is a serious condition, and it requires attention. Despite this major disability, there is limited information on the anatomical location of the GV with the vertebral bodies and psoas muscles. Among the anatomical GV positions, its location between the vertebral body and the iliopsoas muscle is challenging to confirm during surgical procedures in the retroperitoneal space (Figure 1) [11]. There is a risk of GV injury in such cases if the GV is not sufficiently separated from the psoas muscle.

Understanding this relationship can help spine surgeons reduce the risk of GV injury. The current study aimed to investigate the anatomical position of the GV from the viewpoint of spine surgery and to identify the risk factors associated with GV injury. A previous report showed that lumbar scoliosis causes the nerve roots and retroperitoneal vessels to shift relative to the vertebral body [12]. Hence, the spinal alignment and GV position were also analyzed.

## 2. Materials and Methods

### 2.1. Study Design and Setting

**Figure 2:**
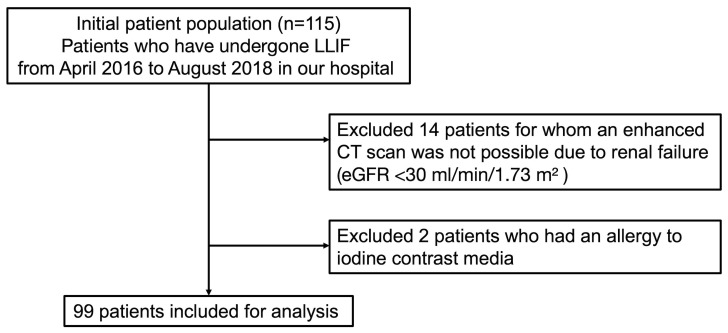
Flowchart of patient selection.

This was a single-center retrospective study. After the research was approved by the ethics committee, we reviewed data on patients who underwent LLIF at our hospital from April 2016 to August 2018. Patients with renal dysfunction or allergy to contrast media used in computed tomography (CT) scans were excluded (Figure 1). Finally, 99 consecutive patients were retrospectively analyzed (38 men and 61 women with an average age of 70.4 ± 8.4 years) (Figure 2).

### 2.2. Classification of GV Location

**Figure 3:**
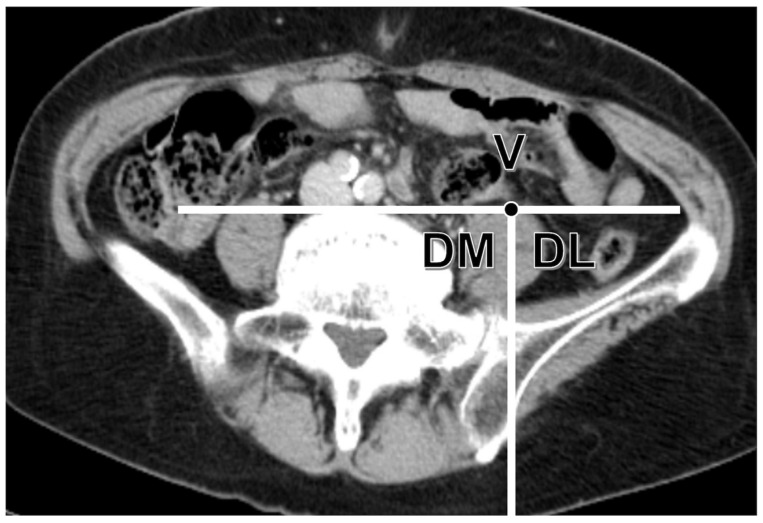
Classification method for ureteral running location. The region was divided into three by drawing a line parallel to the dorsoventral axis from the contact point of the psoas muscle and a tangent line from the ventral side of the lumbar disk to the psoas muscle. V: ventral side, DM: dorsal medial side, DL: dorsal lateral side.

A 64-line multi-slice CT scanner (Light Speed VCT; GE Healthcare Bio-Sciences, Piscataway, NJ, USA) was used, and the GV location at each disc level (L1–L2, L2–L3, L3–L4, L4–L5, and L5–S) was evaluated via contrast-enhanced CT scan. The GV was evaluated in the venous or excretory phase. The levels in which GVs could not be identified in position were omitted. Based on body weight (600 mg L/kg), the contrast material used was either iohexol or iopamidol. The acquisition parameters were as follows: tube voltage, 120 kV; tube current, 120–700 mA; SD, 14.58; and Helical pitch, 1.375 mm. The GV positions were divided into three regions—V: ventral side, DM: dorsal medial side, and DL: dorsal lateral side—in the axial image on the CT scan. To divide the GV positions, a tangent line was drawn from the ventral side of the lumbar disc to the psoas muscle. Another line was drawn parallel to the dorsoventral axis from the contact point of the psoas muscle (Figure 3) [11]. The DM region was surrounded by the vertebral body and psoas muscle. This might be included in the corridor of all LLIF approaches. Hence, the DM region was assumed to be in the most dangerous anatomical position for GV injury.

### 2.3. Patients with GV in the DM Region

The patients were then divided into group M which included those with GV in the DM region at any vertebral level, and group O which included those without GV in DM region at all vertebral levels. Group M was at risk for GV injury during LLIF. The two groups were compared in terms of sex and spinal parameters.

**Figure 4:**
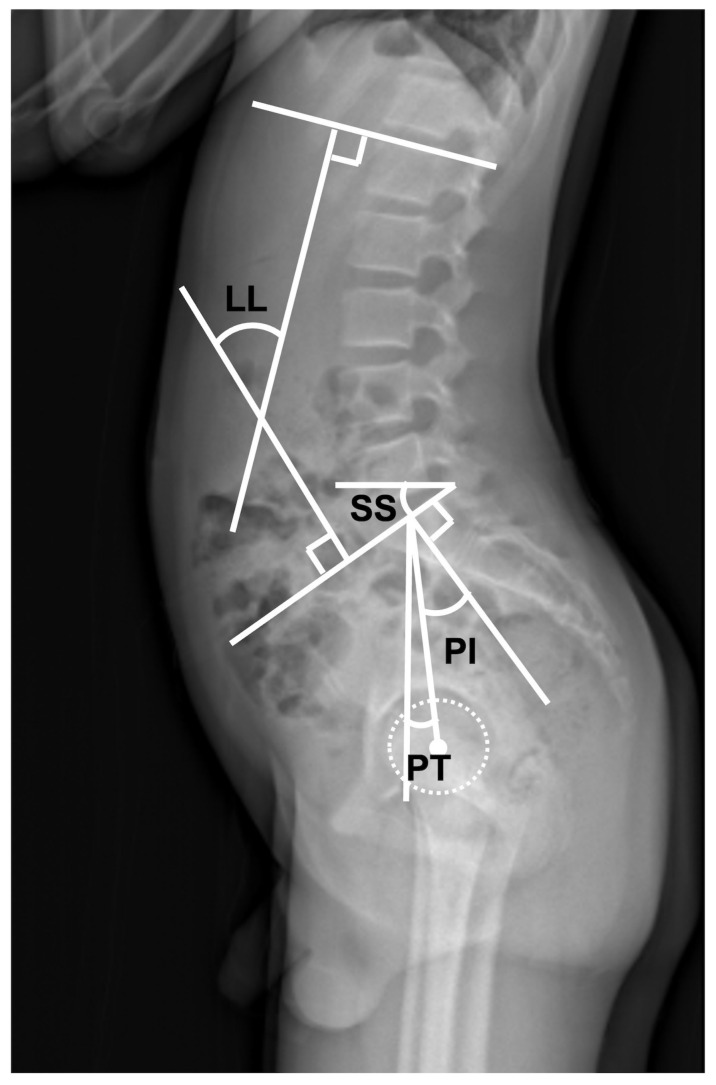
The sagittal spinal parameter. LL: lumber lordosis, PI: pelvic incidence, PT: pelvic tilt, SS: sacral slope.

The EOS imaging system (BioSpace, Paris, France), a slot-scanning X-ray imager, was used to evaluate spinal parameters (LL: lumber lordosis, PI: pelvic incidence, PT: pelvic tilt, SS: sacral slope, and Cobb angle) (Figure 4) [13,14].

### 2.4. Statistical Analysis

Data were presented as mean and standard deviations or numbers and percentages. Categorical data were compared between the two groups using Fisher’s exact test. All analyses of continuous variables were performed using the Mann–Whitney U test. Statistical analyses were conducted using the Easy R (EZR) software version 4.0.3 (Saitama Medical Center, Jichi Medical University, Kawagoeshi, Japan) [15]. A *p* value of <0.05 was considered significant.

## 3. Results

### 3.1. Characteristics of the Patients

The primary diseases were degenerative scoliosis, degenerative spondylolisthesis, fracture, and others in 41, 39, 15, and 4 cases, respectively. The spinal parameters were as follows: LL, 24.0° ± 10.1°; PI, 48.0° ± 10.0°; PT, 30.1° ± 30.9°; and SS, 24.0° ± 9.9°. In the actual surgery, there were no cases of GV injury.

### 3.2. GV Location at the Lumbar Disc Level

**Figure 5:**
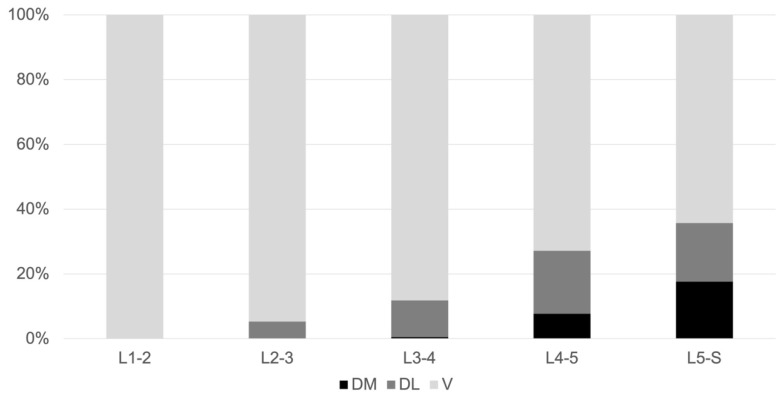
Proportion of gonadal veins at each level.

At the upper lumbar disc levels (L1–L2, L2–L3), the GVs were commonly located ventral to the vertebral body and the psoas muscle (V regions). By contrast, the GVs were located dorsal to the vertebral body and psoas muscle (DM and DL regions) at the lower lumbar disc levels (L3–L4, L4–L5, and L5–S). In particular, in the lower lumbar areas, the GVs were frequently surrounded by the DM region: L3–L4, *n* = 1 (0.5%); L4–L5, *n* = 15 (7.7%); and L5–S, *n* = 34 (17.6%) (Table 1, Figure 5).

### 3.3. Differences in the Right and Side and in Terms of Sex

In the DL group, the GVs were more frequently observed on the left side than on the right side at the L3–L4 level (Table 2). The GVs at the L3–L4 levels (*n* = 2) and each at the L4–L5 levels and the L5–S levels (*n* = 1) on the left side and at the L3–L4 levels (*n* = 1), L4–L5 levels (*n* = 2), and L5–S levels (*n* = 4) on the right side were challenging to identify because of poor contrast enhancement. The GVs located in the DM region were as follows: L4/L5, left: 7.1% and right: 8.2%; L5/S, left: 20.4% and right: 14.7%. Women commonly presented with GVs in the DM region (*p* < 0.01) (Table 3).

### 3.4. Comparison between Group M and Group O

Group M had a higher incidence of degenerative scoliosis (74.1% vs. 29.2%, *p* < 0.01); a significantly larger Cobb angle (16.0° ± 16.4° vs. 5.1° ± 11.3°, *p* < 0.01), PT (30.6 ± 9.3 vs. 21.2 ± 9.1, *p* < 0.01), PI (51.5 ± 10.5 vs. 46.6 ± 9.6, *p* = 0.03), and PI minus LL (29.5 ± 18.6 vs. 16.2 ± 15.5, *p* < 0.01); and a smaller LL (22.0 ± 18.4 vs. 29.7 ± 11.3, *p* = 0.03) than group O (Table 4).

## 4. Discussion

At the upper lumbar levels (L1–L2 and L2–L3), the GVs were commonly located ventral to the vertebral body and psoas muscle (V regions). By contrast, the GVs were located more dorsal (DM or DL regions) at the lower lumbar levels. The proportion of GVs in the dangerous DM region did not differ between the left and right sides. However, the GV was significantly more common in women than in men. Further, the GV in the DM region was associated with degenerative scoliosis and spinal sagittal malalignment.

Previous studies have reported on the GV anatomy. However, the current study was conducted from the viewpoint of spine surgery, and an imaging study of the association between the spine and GV was performed. The GVs are paired structures that drain the gonads (ovaries in women and testes in men). They are paired with the gonadal arteries, and they ascend in the abdomen along the psoas muscle anterior to the ureters [16]. Similar to the suprarenal veins, each side drains differently. In adults, the right testicular vein joins the inferior vena cava at an acute angle, and the left testicular vein enters the left renal vein [17,18]. In addition, the left GVs are longer than the right ones [19]. In the current study, the left GV was present more cranially than the right one which is consistent with the results of previous studies. Anatomically, the ovaries are pelvic organs, and the testes are located more ventrally. Thus, the GV was located closer to the vertebral body in women. Hence, the GV was located in close proximity to the spine and psoas major muscle at the lower levels in women.

Then, we examined which patients could be at risk for GV injury during LLIF. The DM regions surrounded by the psoas major muscle and vertebrae were at the highest risk for GV injury. Actual minor vascular injuries have occurred during OLIF [9], and organs in the DM region that are blind spots for the surgeon are more likely to be injured during the surgical procedure. Results showed that group M had a higher incidence of degenerative scoliosis than group O. Heeren et al. revealed that the overlap between the retroperitoneal vessels and inferior vertebral endplates at the disk level in scoliotic spines varies significantly with the direction of the curvature, level of deformity, and degree of axial rotation [20]. Gilad et al. showed that the risk of vessel injury is further increased by rotational deformity of the spine. However, there were no significant alterations in the relative position of the neurovascular structures in patients with spondylolisthesis [12]. Hence, in female patients with sagittal malalignment and scoliosis, the GV is more common in the DM region, and there is a potential risk of GV injury. Therefore, cautious intraoperative dissection is important when considering LLIF of the lower lumbar spine in female patients with scoliosis.

There is no consensus on the strategies that should be used in the event of an actual GV injury. Although the details are unknown, one case of ovarian venous injury was reported, and it was managed with packing treatment. In some cases, the gonadal arteries were cauterized in urologic and gynecologic cases [21]. In these cases, the gonadal arteries did not deteriorate due to advanced age. Ovarian vein embolization is occasionally performed on women, and its effect on functional prognosis can be minimal [22]. In men, testicular vein injury may cause infertility due to increased testicular temperature caused by venous congestion [23]. Therefore, if LLIF is performed in elderly patients, GV coagulation is unlikely to affect function and ablation may also be considered. In LLIF, the skin incision is small, and its field of view is quite limited. Thus, active intraoperative bleeding from the vessels can significantly disturb the surgical progress. Therefore, spine surgeons must be aware of the presence of GVs and consider the risk of injury preoperatively, even though the response to injury may be relatively easy.

The current study had several limitations. First, a CT scan was performed while in the supine position. Meanwhile, the actual surgical position was the decubitus position. Further, whether retroperitoneal organs move from the supine position to the lateral decubitus position was not considered [24]. Second, the number of patients was relatively small. Third, the proximity of the gonadal veins to other structures, such as neural structures, has not been discussed. Further study will be considered in the future.

## 5. Conclusions

The association between the LLIF corridor and gonadal vessel distribution was examined via a preoperative contrast-enhanced CT scan. In the DM region between the psoas major muscle and vertebral bodies, where there is a risk of injury, the GV was more common in women and at the lower lumbar level (L4–L5). In addition, the incidence of GV was higher in the DM region in patients with scoliosis. Therefore, we should pay attention to the GVs in the preoperative image, and caution is required when performing surgery with LLIF in such cases.

## Figures and Tables

**Table 1:** Distribution of gonadal veins at each level.

	L1–L2 Levels (%)	L2–L3 Levels (%)	L3–L4 Levels (%)	L4–L5 Levels (%)	L5–S Levels (%)
V	31 (100)	160 (94.6)	172 (88.2)	133 (72.9)	124 (64.3)
DM	0	0	1 (0.5)	15 (7.7)	34 (17.6)
DL	0	9 (5.3)	22 (11.3)	38 (19.5)	35 (18.1)

**Table 2:** Differences in gonadal vein distribution between the left and right sides.

	Left Side (%)	Right Side (%)	*p* Value
L1–L2 level			1.0
V	21 (100)	10 (100)	
DM	0	0	
DL	0	0	
L2–L3 level			0.54
V	83 (93.3)	77 (96.2)	
DM	0	0	
DL	6 (6.7)	3 (3.8)	
L3–L4 level			<0.01
V	80 (82.4)	92 (93.9)	
DM	0	1 (1.0)	
DL	17 (17.5)	5 (5.1)	
L4–L5 level			0.07
V	66 (67.3)	76 (78.3)	
DM	7 (7.1)	8 (8.2)	
DL	25 (25.5)	13 (13.4)	
L5–S level			0.12
V	56 (57.2)	68 (71.6)	
DM	20 (20.4)	14 (14.7)	
DL	22 (22.4)	13 (13.7)	

Levels in which the GVs could not be identified were omitted.

**Table 3:** Differences in the gonadal veins in the DM region between male and female participants.

	Men (%)	Women (%)	*p* Value
L3–L4 level	0	1 (0.7)	<0.01
L4–L5 level	0	15 (7.7)	<0.01
L5–S level	1 (0.5)	33 (17.1)	<0.01

**Table 4:** Comparison of demographic characteristics and radiographic parameters between groups M and O.

	Group M *n* = 27	Group O *n* = 72	*p* Value
Age, years, mean (SD)	70.3 (6.2)	70.4 (9.2)	0.95
Sex, male/female, *n*	1/26	37/35	<0.01 *
BMI, kg/m^2^, mean (SD)	22.9 (3.8)	24.3(3.5)	0.06
Cobb angle (°) (SD)	16.0 (16.4)	5.1 (11.3)	<0.01 *
LL (L1–S1 level) (°) (SD)	22.0 (18.4)	29.7 (14.6)	0.03 *
PI (°) (SD)	51.5 (10.5)	46.6 (9.6)	0.03 *
PI–LL (°) (SD)	29.5 (18.6)	16.2 (15.5)	<0.01 *
PT (°) (SD)	30.6 (9.3)	21.2 (9.1)	<0.01 *
SS (°) (SD)	20.9 (11.0)	25.2 (9.4)	0.06
Disease, *n* (%)			<0.01 *
Degenerative scoliosis	20 (74.1)	21 (29.2)	
Degenerative spondylolisthesis	5 (18.5)	34 (47.2)	
Fracture	2 (7.4)	13 (18.1)	
Others	0	4 (5.6)	

Group M: patients with GV in the DM region at any level. Group O: patients without GV in the DM region at all levels. LL: lumbar lordosis, PI: pelvic incidence, PT: pelvic tilt, SS: sacral slope. SD: standard deviation. * *p* < 0.05.

## Data Availability

The datasets generated and/or analyzed during the current study are available from the corresponding author on reasonable request.

## References

[1] Moller D.J., Slimack N.P., Acosta F.L., Koski T.R., Fessler R.G., Liu J.C. (2011). Minimally invasive lateral lumbar interbody fusion and transpsoas approach–related morbidity. Neurosurg. Focus.

[2] Ozgur B.M., Aryan H.E., Pimenta L., Taylor W.R. (2006). Extreme Lateral Interbody Fusion (XLIF): A novel surgical technique for anterior lumbar interbody fusion. Spine J..

[3] Oliveira L.B., Marchi L.M., Coutinho E., Pimenta L. (2010). A Radiographic Assessment of the Ability of the Extreme Lateral Interbody Fusion Procedure to Indirectly Decompress the Neural Elements. Spine.

[4] Rodgers W.B., Gerber E.J.P.-C., Patterson J.B. (2011). Intraoperative and Early Postoperative Complications in Extreme Lateral Interbody Fusion: An analysis of 600 cases. Spine.

[5] Allain J., Dufour T. (2019). Anterior lumbar fusion techniques: ALIF, OLIF, DLIF, LLIF, IXLIF. Orthop. Traumatol. Surg. Res..

[6] Mobbs R.J., Phan K., Malham G., Seex K., Rao P.J. (2015). Lumbar interbody fusion: Techniques, indications and comparison of interbody fusion options including PLIF, TLIF, MI-TLIF, OLIF/ATP, LLIF and ALIF. J. Spine Surg..

[7] Isaacs R.E., Sembrano J.N., Tohmeh A.G., SOLAS Degenerative Study Group (2016). Two-Year Comparative Outcomes of MIS Lateral and MIS Transforaminal Interbody Fusion in the Treatment of Degenerative Spondylolisthesis. Part II: Radiographic findings. Spine.

[8] Shen F.H., Samartzis D., Khanna A.J., Anderson D.G. (2007). Minimally Invasive Techniques for Lumbar Interbody Fusions. Orthop. Clin. N. Am..

[9] Aguirre A.O., Soliman M.A.R., Azmy S., Khan A., Jowdy P.K., Mullin J.P., Pollina J. (2021). Incidence of major and minor vascular injuries during lateral access lumbar interbody fusion procedures: A retrospective comparative study and systematic literature review. Neurosurg. Rev..

[10] Zeng Z.-Y., Xu Z.-W., He D.-W., Zhao X., Ma W.-H., Ni W.-F., Song Y.-X., Zhang J.-Q., Yu W., Fang X.-Q. (2018). Complications and Prevention Strategies of Oblique Lateral Interbody Fusion Technique. Orthop. Surg..

[11] Kagami Y., Nakashima H., Satake K., Ito K., Tanaka S., Segi N., Ouchida J., Morita M., Ando K., Kobayashi K. (2022). Assessment of Ureters at Dangerous Locations in Lateral Lumbar Interbody Fusion. Spine Surg. Relat. Res..

[12] Regev G.J., Chen L., Dhawan M., Lee Y.P., Garfin S.R., Kim C.W. (2009). Morphometric Analysis of the Ventral Nerve Roots and Retroperitoneal Vessels with Respect to the Minimally Invasive Lateral Approach in Normal and Deformed Spines. Spine.

[13] Deschênes S., Charron G., Beaudoin G., Labelle H., Dubois J., Miron M.-C., Parent S. (2010). Diagnostic Imaging of Spinal Deformities: Reducing patients radiation dose with a new slot-scanning X-ray imager. Spine.

[14] Whittle M., Evans M. (1979). Instrument for measuring the cobb angle in scoliosis. Lancet.

[15] Kanda Y. (2013). Investigation of the freely available easy-to-use software ‘EZR’ for medical statistics. Bone Marrow Transplant..

[16] Karaosmanoglu D., Karcaaltincaba M., Karcaaltincaba D., Akata D., Ozmen M. (2009). MDCT of the Ovarian Vein: Normal Anatomy and Pathology. Am. J. Roentgenol..

[17] Szpinda M., Elminowska-Wenda G., Wiśniewski M., Frąckiewicz P. (2005). Morphometric analysis of the gonadal veins in human foetuses. Ann. Anat..

[18] Pai M., Vadgaonkar R., Rai R., Nayak S.R., Jiji P.J., Ranade A., Prabhu L., Madhyastha S. (2008). A cadaveric study of the testicular artery in the South Indian population. Singap. Med. J..

[19] Barber B., Horton A., Patel U. (2012). Anatomy of the Origin of the Gonadal Veins on CT. J. Vasc. Interv. Radiol..

[20] Makanji H.S., Le H., Wood K.B., Jenis L.G., Cha T.D. (2017). Morphometric Analysis of the Retroperitoneal Vessels with Respect to Lateral Access Surgery in Adult Scoliosis. Clin. Spine Surg..

[21] Hsu C.-W., Chang M.-C., Wang J.-H., Wu C.-C., Chen Y.-H. (2019). Incidence and Clinical Outcomes of Gonadal Artery Injury during Colorectal Surgery in Male Patients. J. Gastrointest. Surg..

[22] Pastuszak A.W., Wang R. (2015). Varicocele and testicular function. Asian J. Androl..

[23] Joh M., Grewal S., Gupta R. (2021). Ovarian Vein Embolization: How and When Should It Be Done?. Tech. Vasc. Interv. Radiol..

[24] Ouchida J., Kanemura T., Satake K., Nakashima H., Segi N. (2019). Anatomic evaluation of retroperitoneal organs for lateral approach surgery: A prospective imaging study using computed tomography in the lateral decubitus position. Eur. Spine J..

